# Psychosis secondary to indomethacin. A case report

**DOI:** 10.1192/j.eurpsy.2021.1747

**Published:** 2021-08-13

**Authors:** S. Santiago González, C. Gómez Sánchez-Lafuente, A. Martinez Rico, J. Hurtado Molina

**Affiliations:** 1 Psychiatry, Malaga Regional Hospital, Málaga, Spain; 2 General Practice, Malaga District, Málaga, Spain

**Keywords:** Indomethacin, COVID-19

## Abstract

**Introduction:**

Indomethacin, a non-steroidal anti-inflammatory treatment used in various inflammatory diseases, is one of the drugs that has been related to the appearance of psychotic symptoms as a side effect.

**Objectives:**

Point out the importance of knowing the possible psychiatric symptoms that some drugs can cause as a side effect.

**Methods:**

Description of a clinical case and bibliography review.

**Results:**

We present the case of a 71-year-old woman, with no previous mental health history, who is referred by her primary care physician due to the presence of auditory hallucinations and self-referential ideas. As a somatic history, the patient presented Rheumatoid Arthritis under control by rheumatology and acoustic neuroma, under control by neurosurgery. Treatment with Risperidone was started, up to 2 mg, which helped control her symptoms. After an exhaustive study of her situation, the possibility that her symptoms were a side effect of her usual treatment was raised. It was evidenced that the patient had taken a higher dose of Indomethacin than prescribed by the rheumatologist, reason why its daily intake was suspended, and subsequently an improvement and even suppression of symptoms was seen. Later, due to a misunderstanding, the drug was reintroduced, and symptoms appeared again.

**Conclusions:**

The appearance of psychotic symptoms has been related to the intake of various drugs, including Indomethacin. It is essential to carry out a differential diagnosis if psychotic symptoms appear in the subject.

**Disclosure:**

No significant relationships.

